# Polymorphisms in the Promoters of the *MMP-2* and *TIMP-2* Genes Are Associated with Spontaneous Deep Intracerebral Hemorrhage in the Taiwan Population

**DOI:** 10.1371/journal.pone.0142482

**Published:** 2015-11-09

**Authors:** Yi Chun Chen, Wei Min Ho, Yun Shien Lee, Huei Wen Chen, Chiung-Mei Chen

**Affiliations:** 1 Department of Neurology, Chang Gung Memorial Hospital Linkou Medical Center and College of Medicine, Chang-Gung University, Taoyuan, Taiwan; 2 Department of Biotechnology, Ming Chuan University, Taoyuan, Taiwan; 3 Genomic Medicine Research Core Laboratory, Chang Gung Memorial Hospital, Taoyuan, Taiwan; INRS, CANADA

## Abstract

**Background:**

Spontaneous intracerebral hemorrhage (ICH) is a devastating stroke subtype. Matrix metalloproteinases (MMPs) function in the degradation of extracellular matrix and the activities of MMPs are modulated by their endogenous inhibitors, tissue inhibitors of metalloproteinases (TIMPs). This study aimed to discuss relationship of *MMP-2* and *TIMP-2* to spontaneous deep ICH (SDICH) susceptibility and hematoma size.

**Methods:**

Associations were tested by logistic regression and general linear models (GLM) where appropriate, adjusting with covariables of age, sex, hypertension, diabetes mellitus, smoking, and alcohol consumption. Association analyses were performed first by stratification of genders and then by the age of 65 years old (y/o). Elder population was defined as subjects who were older than 65 y/o.

**Results:**

There were 396 SDICH patients and 376 control subjects in this study. In the elder group, rs7503607 C>A variant in *TIMP-2* was associated with SDICH in male and overall patients (OR = 3.49, 95% CI 1.45 to 8.40, P = 0.005 and OR = 2.45, 95% CI 1.37 to 4.38, P = 0.003, respectively) in additive genetic model. In recessive genetic model, rs2285053 TT genotype in *MMP-2* was correlated to SDICH in male patients and overall elder group (OR = 7.30, 95% CI 1.3 to 40, P = 0.02 and OR = 2.91, 95% CI 1.02 to 8.31, P = 0.046, respectively), and rs7503726 AA genotype in *TIMP-2* was associated with SDICH in female patients (OR = 0.29, 95% CI 0.1 to 0.84, P = 0.02). In younger male and overall younger patients, SDICH patients who had supratentorial hemorrhage had significantly lower frequency of AA genotypes in rs7503726 than those with infratentorial hemorrhage (OR = 0.36, 95% CI 0.17 to 0.75, P = 0.006 and OR = 0.43, 95% CI 0.22 to 0.84, P = 0.014, respectively). Hemorrhage size increased by 9.7 (95% CI 2.1 to 43, P = 0.004) cm^3^ per minor allele (A) of the rs7503607 variant in the elder female patients and increased by 4.3 (95% CI 1.4 to 12.9, P = 0.009) cm^3^ per minor allele (A) in all elder patients. In younger patients, the hemorrhage size decreased by 3.3 (95% CI 1.2 to 9.5, P = 0.03) cm^3^ per minor allele of the s7503726 variant in the female patients.

**Conclusions:**

This study showed a significant association between the variants of *MMP-2* and *TIMP-2* promoters and SDICH susceptibility with significant age and gender differences. Hemorrhage location and size might be affected by *TIMP-2* promoter variants in the SDICH patients.

## Introduction

Spontaneous intracerebral hemorrhage (ICH) is a common devastating stroke subtype that is associated with high morbidity and mortality despite the improvement in neurological intensive care [1, 2]. In contrast to ischemic stroke, ICH leads to higher rates of 30-day mortality (17.9% versus 4%) and disability (78.7% versus 61.2%) in Taiwan [3]. In acute stage, mortality is determined by the initial Glasgow Coma Scale (GCS) and hemorrhage size [4]. Hypertension is the major risk factor of ICH, which accounts for about 54% of ICH cases [5]. When hypertension persists, proliferation of smooth muscle cell (SMC) in cerebral arterioles may occur, with the formation of reactive hyperplasia replaced by collagen tissues at vascular walls [6]. When the deposition of collagen is insufficient, arteriolar wall will dilate to become Charcot-Bouchard aneurysm and weaken the vascular integrity [7]. Bleeding of the aneurysm is partly determined by the extent of the vascular pathological changes of the SMC and the collagen contents [6].

Genetic predisposition of several pathological pathways to the susceptibility to ICH risks has been speculated [8, 9]. Matrix metalloproteinases (MMPs) pathway has been shown to play multiple roles in tissue remodeling and inflammation reactions in spontaneous ICH [10, 11]. MMPs are a family of zinc/calcium containing endopeptidases which function in the degrading and remodeling of extracellular matrix (ECM) for their ability of breaking matrix integrity [12]. Gelatinases, a subcategory of MMPs, is particularly unique in blood-brain barrier (BBB) damage due to their ability to digest ECM components, such as elastin and type IV collagen [13]. In this group, gelatinase A (MMP-2) is capable of degrading basement membrane of ECM and contributes to a hemorrhagic phenotype [14]. MMP-2 expression in brain was associated with endothelial cells and reactive astrocytes [15]. The functions of MMPs are partly regulated by the endogenous tissue inhibitors of metalloproteinases (TIMPs) [11]. Certain combinations between MMPs and TIMPs have been reported, in which TIMP-2 is the main endogenous inhibitor of MMP-2 [11, 16]. A recent report suggested that MMP-2 is able to delay the toxicity of β-amyloid (Aβ) species for endothelial cells through breaking down the Aβ structure [14]. However, during the same process, these protective effects might also compromise the integrity of BBB and precipitate a hemorrhagic phenotype. In Aβ-damaged vessels, the activation of MMP-2 in chronic microbleeds and acute ICH was believed to be responsible for triggering the eventual bleeding [15]. In addition, pro-MMP-2 in vitro increased gelatinolytic activity in cerebral aneurysms [17]. One study has shown genetic variation of *TIMP-2* promoter increased ICH risks, suggesting that the MMP-TIMP pathway may play a role in the susceptibility to ICH risks [18].

Because ICH is a heterogeneous disease entity, the study herein focused on spontaneous deep ICH (SDICH). Recently, we showed associations of gelatinase B (MMP-9) and TIMP-1 with SDICH risk with age difference in the Taiwan population [19]. To our knowledge, there is no report addressing the association and interactions of *MMP-2* and *TIMP-2* polymorphisms with SDICH risk and hemorrhage size. Given the *a priori* evidence of MMPs associated with spontaneous ICH susceptibility, this study evaluated whether the genetic variations of promoters in *MMP-2* rs2285053 (-735 C>T), *TIMP-2* rs7503607 (-269 C>A), and *TIMP-2* rs7503726 (-261 G>A) would predispose to SDICH, affect hemorrhage size, and modify the 30-day outcome in the Taiwan population. Here we also reviewed literatures addressing genetic variants of MMPs and TIMPs with ICH risks.

## Methods

### Subjects

Subjects were recruited from acute ward and outpatient clinic of the Department of Neurology, Chang Gung Memorial Hospital (CGMH), Linkou Medical Center during 2008 to 2014. SDICH was diagnosed according to the clinical presentations and brain computed tomography (CT) as previously described [19]. Patients suffering from traumatic or secondary ICH (vascular anomaly or tumor, aspirin, other anti-platelet or anticoagulants use, abnormal platelet count, prolonged prothrombin time or activated partial thromboplastin time) were excluded [19]. Participants were enrolled if he or she agreed to provide a written informed consent. When a patient had compromised capacity because of altered consciousness (GCS <15), the written informed consent was provided by the patient’s legal representative based on the laws and regulations in Taiwan [19]. Participants of the control group were enrolled from subjects who had no history of neurodegenerative diseases, inflammatory and autoimmune diseases, malignancy, and stroke. Controls were selected to match age and sex compared to the SDICH group. This study was approved by the Institutional Review Board of CGMH.

### Clinical Information and definitions

Anthropometric data, including history of hypertension, diabetes mellitus (DM), cigarette using and alcohol consumption, were collected from all participants. Hypertension was diagnosed when blood pressure (BP) repeatedly exceeded 140 mm Hg (systolic) and/or 90 mm Hg (diastolic) or when a participant was taking antihypertensive medications as previously described [19]. DM was defined according to World Health Organization (WHO) criteria [20]. Body mass index (BMI) was calculated by weight in kilograms divided by squared height in meters. Alcohol consumption was defined as drinking ≥210 g per week. Smoking was defined as former or current smoking [21].

All patients received brain CT upon admission to identify the presence of acute ICH and its size and location. Hematomas located at the basal ganglia and thalami were defined as supratentorial hematoma, while those at brainstem and cerebellar hemorrhage were infratentorial. Volume of the hematoma was calculated using the so-called ABC method based on CT of brain [22]. Short term outcome was assessed with the modified Rankin scale (mRS) on day 30 after SDICH events. Subjects who were dead or dependent (mRS > 3) at the 30 day follow-up were considered poor prognosis [23].

### Selection of SNP and Genotyping

The cytogenetic location of *MMP-2* was at 16q12.2. In the promoter variant of *MMP-2*, we examined rs2285053 (-735 C>T) which has been shown to influence expression and a subsequent increase in *MMP-2* transcription [24]. For *TIMP-2* gene (cytogenetic location at 17q25.3), we selected *TIMP-2* rs7503726 (-261 G>A), which has been discussed in the prior researches addressing spontaneous ICH [18] and cerebral aneurysm [25]. We also looked at rs7503607 (-269 C>A) which is 8 base pairs away from rs7503726 and has not been examined previously.

Blood samples were collected for single nucleotide polymorphism (SNP) genotyping and DNA was isolated from peripheral leukocytes using DNA Extraction kit (Stratagene). The genotype of *MMP-2* rs2285053 was determined according to a matrix-assisted laser desorption/ionization time-of-flight (MALDI-TOF)-based mini-sequencing genotyping method. Sizes of the forward primer (CAGTGGGGTCTTTGTGACCT), reverse primer (GCGTTAGAGACGTTGGAACC), mini-sequencing primer (MSP) (TGACCGAGAATGCGGAC) and the product were 172 base pairs. *TIMP-2* rs7503726 and rs7503607 were analyzed by amplification of a high GC content region which was achieved by two rounds of polymerase chain reaction (PCR) with Pfu enzymatic system. The length of amplified fragments containing the sites was 208 base pairs, which was then genotyped by bi-directional Sanger sequencing.

### Statistical analysis and power estimation

The Pearson’s χ^2^-test or t-test was utilized to compare demographic data and the distributions of genotypes between controls and cases. Two-tailed P-values were derived from the χ^2^-test or Fisher's exact test. Association analyses were performed first stratified by genders and then stratified by the age of 65 y/o. Elder population was defined as subjects ≥ 65 y/o. Multivariable logistic regression was used to analyze the phenotype-genotype associations of SDICH under additive and recessive genetic models. Covariables included age, sex, hypertension, DM, smoking, and alcohol consumption. Hemorrhage size was logarithmic transformed to fit normal distribution [26]. Associations between hematoma size and each of the SNPs were examined by general linear models (GLM) adjusting for age, sex, hypertension, DM, smoking, and alcohol consumption.

We evaluated the ability of detecting an association between a SNP and SDICH by power calculation implemented in QUANTO version 1.0 [27]. In the present case-control study, at the 5% significance level, we had power greater than 0.8 to identify an association under a dominant genetic model when the per-allele genetic effect was greater than an odds ratio of 1.6 for each of the selected SNPs. Analyses were performed using SAS software version 9.1.3 (SAS Institute, Cary, NC, USA). To examine interaction effects of *MMP-2* and *TIMP-2* to SDICH susceptibility, the multiplicative term of genotypes were evaluated in the same models as the interaction term.

## Results

### Patient recruitment

There were 396 SDICH patients and 376 control subjects in this study, including 276 SDICH patients and 211 control subjects in men and 120 SDICH patients and 165 control subjects in women. For both genders, proportion of hypertension history is significantly higher in the SDICH patients than that in the controls (P < 0.0001) (Table 1). Alcohol and cigarette were more frequently used in the male SDICH patients (P < 0.0001). The proportion of DM increased significantly in the female patients compared to that in the female controls (P = 0.01). There was no significant difference in BMI and levels of total cholesterol and triglyceride between patients and controls. Regarding hematoma location in the SDICH patients, proportion of supratentorial hemorrhage tended to be higher in women than in men (P = 0.06). There was no difference regarding hemorrhage size, 30-day mortality and dependent rate (mRS > 3) and 3-year stroke recurrent rate between genders in the SDICH patients.

**Table 1:** Demographic data in patients with spontaneous deep intracerebral hemorrhage and controls.

	Males (n = 487)			Females (n = 285)			Between genders
	SDICH^a^	Controls	P-Value	SDICH^a^	Controls	P-Value	P-Value
	n = 276	n = 211		n = 120	n = 165		
Age (years)	57.5 ± 13.3	59.8 ± 13.2	0.06	63.5 ± 12.1	61.0 ± 9.0	0.06	
Hypertension (%)	86.6	48.8	<0.0001	93.3	51.5	<0.0001	
Diabetes mellitus (%)	15.6	16.2	0.87	27.5	15.4	0.01	
Alcohol use (%)	39.9	19.4	<0.0001	4.2	1.2	0.11	
Smoke (%)	55.8	34.6	<0.0001	5.8	1.8	0.07	
Body mass index (kg/m^2^)	25.2 ± 4.2	25.6 ± 3.4	0.32	24.5 ± 3.8	25.3 ± 3.7	0.11	
Total cholesterol (mg/dL)	171.7 ± 44.4	165.6 ± 56.1	0.19	178.6 ± 47.9	177.0 ± 62.1	0.80	
Triglyceride (mg/dL)	141.9 ± 92.4	147.0 ± 99.7	0.60	135.6 ± 72.6	144.6 ± 76.5	0.35	
Supratentorial %	75.7			84.2			0.06
Hemorrhage size (ml)	17.4 ± 19.7			14.1 ± 16.1			0.17
30-day mRS>3 (%)	40.2			40.8			0.91
30-day death (%)	3.9			2.8			0.56
3-y recurrent stroke (%)	13.5			13.7			0.97

Data are expressed as percentage or mean ± SE. Comparisons between controls and SDICH cases were analyzed by χ^2^ test or t-test where appropriate. To convert mg/dL to mmol/L, multiply cholesterol values by 0.02586 and triglycerides by 0.011.

30-day mRS: modified Rankin scale (mRS) on day 30 after SDICH events.

SDICH^a^: spontaneous deep intracerebral hemorrhage.

### Genotype frequency and association analysis of controls and patients

All of the three SNPs were in Hardy-Weinberg equilibrium (significance level of 0.01) in each of the case and control groups. The minor allele frequency (MAF) was 27.3% of *MMP-2* rs2285053, 15.8% of *TIMP-2* rs7503607, and 49.7% of *TIMP-2* rs7503726 in all of the subjects. We identified no association between hypertension and each of the SNPs. Genotyping data are available in S1 Appendix.

Frequency and association of each genotype in SDICH and control subjects were shown in Table 2. Distribution of rs2285053 genotypes suggested a possible recessive model of genetic effect on SDICH in the elder male group. Specifically, in recessive genetic model, frequency of homozygous minor allele (TT) in rs2285053 in *MMP-2* increased significantly in the elder male patients (OR = 7.30, 95% CI 1.30 to 40.00, P = 0.02) and in the overall elder patients (OR = 2.91, 95% CI 1.02 to 8.31, P = 0.046). We did not discover association between rs2285053 and SDICH in additive model. Decreased frequency was observed in homozygous minor allele (AA) in rs7503726 in elder female SDICH patients (OR = 0.29, 95% CI 0.10 to 0.84, P = 0.02) compared to controls, suggesting a protective effect of rs7503726 variation in recessive model. In contrast, when compared to the controls, *TIMP-2* rs7503607 C>A variant occurred more frequently in an additive fashion in the elder male patients (OR = 3.49, 95% CI 1.45 to 8.40, P = 0.005) and in the overall elder patients (OR = 2.45, 95% CI 1.37 to 4.38, P = 0.003). There was no significant association between the SNPs and SDICH in the younger groups.

**Table 2:** Frequencies and associations of the genotypes in patients with spontaneous deep intracerebral hemorrhage and controls.

	Males			Females			All
	SDICH^a^ (%)	Controls (%)	OR^b^ (95% CI^c^),	SDICH (%)	Controls (%)	OR (95% CI),	OR (95% CI),
	n = 276	n = 211	P-Value	n = 120	n = 165	P-Value	P-Value
**Age ≥65 y/o**	n = 83	n = 78		n = 61	n = 54		
*MMP2*							
rs2285053 CC/CT/TT	56.6/30.1/12.1	52.6/44.9/2.6	7.3 (1.3,40), 0.02^2^	47.5/41/9.8	53.7/38.9/7.4	NS	2.91 (1.02,8.31), 0.046^2^
*TIMP2*							
rs7503607 CC/CA/AA	68.7/30.1/1.2	87.2/12.8/0	3.49 (1.45,8.40), 0.005^1^	59/39.3/1.6	68.5/31.5/0	NS	2.45 (1.37,4.38), 0.003^1^
rs7503726 GG/GA/AA	30.1/50.6/19.3	23.1/56.4/20.5	NS^d^	32.8/55.7/11.5	24.1/50/25.9	0.29 (0.10,0.84), 0.02^2^	NS
**Age <65 y/o**	n = 193	n = 133		n = 59	n = 111		
*MMP2*							
rs2285053 CC/CT/TT	52.9/37.8/9.3	50.4/41.4/6.8	NS	57.6/32.2/10.2	55/39.6/5.4	NS	NS
*TIMP2*							
rs7503607 CC/CA/AA	66.8/31.1/2.1	72.9/25.6/1.5	NS	66.1/30.5/3.4	69.4/28.8/1.8	NS	NS
rs7503726 GG/GA/AA	27.5/48.2/24.4	25.6/42.1/32.3	NS	23.7/52.5/23.7	25.2/55/19.8	NS	NS

Data are expressed as homozygous major allele/heterozygous/ homozygous minor allele. Analysis was performed by logistic regression under ^1^additive genetic model and ^2^recessive genetic model and adjust for age, sex, hypertension, DM, alcohol drinking, smoking.

SDICH^a^: spontaneous deep intracerebral hemorrhage

OR^b^: Odds ratio

CI^c^: confidence interval

NS^d^: non-significant

### Association analysis of SNPs and hemorrhage location and size

Associations between SNPs and hemorrhage location were further analyzed in SDICH patients (Table 3). We found that younger male SDICH patients who had supratentorial hemorrhage had significantly lower frequency of AA genotype in rs7503726 than those with infratentorial hemorrhage (OR = 0.36, 95% CI 0.17 to 0.75, P = 0.006). The hemorrhage location was not associated with the other two SNPs.

**Table 3:** Frequencies and associations of *TIMP2* rs7503726 in patients with spontaneous deep intracerebral hemorrhage.

	Males			Females			All
	Supratentorial	Infratentorial	OR^a^ (95% CI^b^),	Supratentorial	Infratentorial		OR (95% CI),
	%	%	P-Value	%	%	P-Value	P-Value
**Age ≥65 y/o**	n = 60	n = 23		n = 50	n = 11		
rs7503726 GG/GA/AA	16.7/53.3/30	26.1/43.5/30.4	NS^c^	34/58/8	27.3/45.5/27.3	NS	NS
**Age <65 y/o**	n = 149	n = 44		n = 51	n = 8		
rs7503726 GG/GA/AA	30.2/50.3/19.5	18.2/40.9/40.9	0.36 (0.17,0.75), 0.006	23.5/52.9/23.5	25/50/25	NS	0.43 (0.22,0.84), 0.014

Data are expressed as percentages of homozygous major allele/heterozygous/ homozygous minor allele of rs7503726 G>A.

Analysis was performed by logistic regression under recessive genetic model and adjusted for age, sex, hypertension, DM, alcohol drinking, and smoking.

^a^OR: Odds ratio

^b^CI: confidence interval

^c^NS: non-significant

Furthermore, we used the GLM to analyze the associations between hematoma size and SNPs (Table 4). The beta values shown in Table 4 indicate the per-allelic effect of minor allele in each SNP on hemorrhage size. We found, in the elder females, patients carrying rs7503607 CA genotype had larger hematoma size (20.31 ± 22.99 cm^3^) than the non-carriers (CC genotype, 8.77 ± 6.20 cm^3^) with a beta value 9.7 (95% CI 2.1 to 43) cm^3^, P = 0.004). In contrast, younger female patients carrying rs7503726 minor allele A had smaller hematoma size (GG: 19.72 ± 22.37 cm^3^, GA: 12.17 ± 11.04 cm^3^ and AA: 9.92 ± 9.13 cm^3^) with a beta value 3.3 (95% CI 1.2 to 9.5) cm^3^, P = 0.03. There were no associations between SNPs and DM, HTN, alcohol or smoking. Furthermore, the two genes had no interaction effect on SDICH susceptibility. We did not find significant effect of *TIMP-2* and *MMP-2* SNPs on the 30-day short term outcome (30-day mRS>3 and 30-day death rate) and the 3-year recurrence.

**Table 4:** Differences in hemorrhage size as a function of the presence or absence of *MMP-2* and *TIMP-2* minor allele in patients with spontaneous deep intracerebral hemorrhage.

	Males		Females		All	
	beta (95% CI^a^) cm^3^	P-Value	beta (95% CI) cm^3^	P-Value	beta (95% CI) cm^3^	P-Value
**Age ≥65 y/o**						
*MMP2*						
rs2285053 C>T	-1.1 (-3.3, 2.8)	NS^b^	-1.3 (-4.8, 2.9)	NS	-1.1 (-2.5, 2)	NS
*TIMP2*						
rs7503607 C>A	3.1 (-1.7, 16)	NS	9.7 (2.1, 43)	0.004	4.3 (1.4, 12.9)	0.009
rs7503726 G>A	1.24 (-3.6, 2.4)	NS	2.5 (-9, 1.5)	NS	-1.5 (-3.2, 1.6)	NS
**Age <65 y/o**						
*MMP2*						
rs2285053 C>T	-1.2 (-2.2, 1.5)	NS	-1.0 (-3.9, 3.6)	NS	-1.2 (-2.1, 1.4)	NS
*TIMP2*						
rs7503607 C>A	-2 (-4.2, 1.1)	NS	2.4 (-1.6, 9.2)	NS	-1.3 (-2.5, 1.5)	NS
rs7503726 G>A	-1.0 (-1.7, 1.7)	NS	-3.3 (-1.2, -9.5)	0.03	-1.2 (-2, 1.3)	NS

Beta (95% CI) derived from GLM models that included tradition risk factors as independent variables show the per-allelic effect of minor allele in each SNP on hemorrhage size.

CI^a^: confidence interval

NS^b^: non-significant

## Discussion

This is the first report demonstrating associations of the variants of *MMP-2* and *TIMP-2* promoters with SDICH susceptibility with significant age and gender differences. In the elderly men, we found that TT genotype of *MMP-2* rs2285053 C>T and A allele of *TIMP-2* rs7503607 C>A were susceptible to SDICH risk. In the elderly women, however, AA genotype of *TIMP-2* rs7503726 G>A was a protective factor for SDICH. In the younger male patients with SDICH, subjects with infratentorial hemorrhage tended to have AA genotype of rs7503726 compared to those with supratentorial hemorrhage, suggesting different pathophysiology of SDICH in different loci. In addition, *TIMP-2* promoter variants were associated with hemorrhage size in female patients, in which rs7503607 variant increased hemorrhage size while rs7503726 variant decreased it. These associations were independent of traditional risk factors and had no interaction with environmental factors.

Prior reports and our study addressing MMPs/TIMPs showed inconsistent findings between studies regarding all of the studied variants (Table 5). While a prior study suggested that AA genotype of rs7503726 G>A was at increased risk of developing ICH in a genetic recessive model in a Germany population [18], the study herein showed a contradictory result. This study demonstrated AA genotype of *TIMP-2* rs7503726 variant was a protective factor of SDICH in the elderly female population. The disparity between the two reports may be due to different ethnicity with different MAF (39.14% of 7503726 in prior study and 49.7% in ours), heterogeneity of phenotypes in the prior study (including lobar and deep ICH), different epigenetic regulation, and different study designs. Further large-scale investigation is needed for clarification.

**Table 5:** Prior association studies of MMPs/TIMPs with ICH risks.

	SNP	Ethnicity	Number	Effect of minor allele	Reference
MMP-9	rs3918241, rs1805088, rs17576, rs3918254, rs3787268, rs17577	Chinese Han	181 ICH patients/197 hypertensive controls	No association	Yang et al.[28]
	rs3918242, rs17576, rs3787268, rs2250889	Taiwanese	326 deep ICH/439 controls	rs3787268: protective in the elderly. rs2250889: protective in the younger males	Ho et al.[19]
TIMP-1	rs4898, rs2070584	Chinese Han	410 ICH/305 controls	rs2070584: ICH risk in males	Wang et al.[29]
	rs4898	Taiwanese	326 deep ICH/439 controls	rs4898: protective in the elder males	Ho et al.[19]
MMP-2	rs2285053	Taiwanese	396 deep ICH/376 controls	rs2285053: ICH risk in males	Present study
TIMP-2	rs7503726	German	45 ICH/253 controls	rs7503726: ICH risk	Reuter et al.[18]
	rs7503607, rs7503726	Taiwanese	396 deep ICH/376 controls	rs7503607: ICH risk rs7503726: protective in females	Present study
MMP-3	rs520540, rs602128, rs679620	Korean	70 ICH patients/401 controls	No association	Kim et al.[30]

Expression of human *MMP-2* is mostly regulated through cis-acting regulatory elements in the promoter [31]. The constitutive and induced expression of MMP-2 is therefore subjected to regulation by transcription factors and affected by SNP in promoter [32]. *MMP-2* rs2285053 C allele has higher promoter activity and mRNA expression than T allele. T allele of rs2285053 might impair Sp1-type promoter motif (CCACC box) binding element and alter *MMP-2* transcription, leading to lower promoter activity [24]. Therefore, it is postulated that T allele might be a protective factor for ICH. However, we found that rs2285053 T allele was a risk of SDICH in the elderly male population, we suggested that the pathophysiology underlying the association might be pleiotropic and beyond the proteolytic effect of MMP-2.

Vascular SMC might be compromised by hypertension, which may further cause ECM degradation [33]. The role of age difference in MMPs and TIMPs pathway on cerebral aneurysms has been addressed in a prior animal model, in which quantitative PCR showed an increase of TIMP-2 mRNA in the early stage of aneurysm progression but not in the late stage, whereas mRNA expression of MMP-2 increased in the late stage [25]. Progressively declined MMP-2 and TIMP-2 baseline level and increasing MMP-9 level after ICH were observed *in vivo* study [10]. This temporal effect suggested possible epigenetic mechanism in the regulations of *MMP-2* and *TIMP-2*. Due to the high GC content in the promoters of *TIMP-2*, DNA methylation may also contribute to the changes in serum level of TIMP-2. Imbalance between MMP-2 and TIMP-2 in the late stage of vascular damage may be responsible for ECM degradation leading to the progression and rupture of damaged vessels. In addition, estrogen was related to nitric oxide production and inflammation pathway, which may influence MMP-2 expression in the female subjects and contribute to gender difference in the associations of MMP-2 with phenotypes [11, 34]. Further functional study for confirmation is needed. Molecules regulating MMP/TIMP activities have been reported for the past decades. One of these medicines is angiotensin converting enzyme (ACE) inhibitors [35]. Angiotensin II plays a major role in vascular remodeling and has been shown to increase TIMP-2 expression in rat aortic SMC *in vivo* [36]. In clinical studies, reduction in plasma MMP-2 and MMP-9 and increase in TIMP-1 were shown to response to anti-hypertensive treatment with angiotensin converting enzyme (ACE) inhibitors or angiotensin receptor blockers [37–39]. Left ventricular remodeling following acute myocardial infarction was also shown to be suppressed by decrease in MMP-9 level via inhibition of the renin-angiotensin system [39]. Our group has demonstrated that ACE T-D haplotype constructed by A-240T and Alu I/D was associated with female SDICH. The association was via the effect of hypertension. The finding was replicated by two meta-analysis studies [40–42]. Taken together, these studies suggest polymorphisms in MMPs/TIMPs and their regulator, ACE, are associated with SDICH.

Volume of hematoma determines the clinical prognosis of spontaneous ICH. Previous reports showed inconsistent change of TIMP-2 protein levels in responding to ICH, in which TIMP-2 was decreasing in acute phase of ICH (7 days) [43], while increasing in the other report [44] and not changed in another [18]. Increasing TIMP-2 level was noted over 14 days in an animal model [45]. Significant correlations between serum levels of MMP-2 and TIMP-2 were shown in normal subjects and after spontaneous ICH [43]. There was also evidence showing that serum levels of MMP-2 and TIMP-2 were highest at baseline in ICH patients while MMP-9 and TIMP-1 were highest at 24 hours, in which study, baseline MMP-9 was positively correlated to perihematomal edema volume and its inhibitor TIMP-1 was negatively correlated to perihematomal edema volume [43]. All of these suggested expressions of *MMP*s and *TIMP*s are associated with spontaneous ICH. This study suggested that hemorrhage size might be affected by *TIMP-2* promoter variants in the SDICH patients.

This is the first study proposing that *MMP-2* and *TIMP-2* genotypes are associated with the SDICH susceptibility and hematoma size with age and gender difference. Utilizing genetic approach, we found multiple genetic factors associated with SDICH risks, including genes involved in inflammation [46], lipid metabolism [47], hypertension [48], and ECM integrity [19]. Among these genes, *APOE* and MMP-9 pathway genes are especially important in the interaction with alcohol consumption, one of the major environmental risk factor of SDICH. The strength of our studies however is limited by small sample size. Under the present female cases, genetic effect that was less than 1.5 may not be identified in this study. In addition, because the patients were approached only when they matched the inclusion/exclusion criteria of enrollment, it forbade us to estimate the exact percentage of our patients among overall cases admitted to our hospital during ascertainment. Another limitation of this study is that the SDICH patients were recruited from the Department of Neurology, which might cause a smaller average hemorrhage size compared to those admitted to the Department of Neurosurgery. In addition, this study did not perform brain CT on the normal controls, which again forbade us to exclude cerebral aneurysm or microbleedings in the normal subjects. Further replicated study is needed to confirm the results herein, especially for the female population to avoid a false negative result.

## Conclusion

This study showed a significant association between the variants of *MMP-2* and *TIMP-2* promoters and SDICH susceptibility with significant age and gender differences. Hemorrhage location and size might be affected by *TIMP-2* promoter variants in the SDICH patients. These associations were independent of traditional risk factors and had no interaction with environmental factors.

## Supporting Information

S1 Appendix Genotyping data. (XLS)
